# The importance of context: When relative relief renders pain pleasant

**DOI:** 10.1016/j.pain.2012.11.018

**Published:** 2013-03

**Authors:** Siri Leknes, Chantal Berna, Michael C. Lee, Gregory D. Snyder, Guido Biele, Irene Tracey

**Affiliations:** a Centre for Functional Magnetic Resonance Imaging of the Brain (FMRIB), Nuffield Department of Clinical Neurosciences (Nuffield Division of Anaesthetics), University of Oxford, Oxford OX3 9DU, UK; b Department of Psychology, University of Oslo, 0317 Oslo, Norway

**Keywords:** Reward, Neuroimaging, Dread, Relief, Value

## Abstract

Context can influence the experience of any event. For instance, the thought that “it could be worse” can improve feelings towards a present misfortune. In this study we measured hedonic feelings, skin conductance, and brain activation patterns in 16 healthy volunteers who experienced moderate pain in two different contexts. In the “relative relief context,” moderate pain represented the best outcome, since the alternative outcome was intense pain. However, in the control context, moderate pain represented the worst outcome and elicited negative hedonic feelings. The context manipulation resulted in a “hedonic flip,” such that moderate pain elicited positive hedonics in the relative relief context. Somewhat surprisingly, moderate pain was even rated as *pleasant* in this context, despite being reported as painful in the control context. This “hedonic flip” was corroborated by physiological and functional neuroimaging data. When moderate pain was perceived as pleasant, skin conductance and activity in insula and dorsal anterior cingulate were significantly attenuated relative to the control moderate stimulus. “Pleasant pain” also increased activity in reward and valuation circuitry, including the medial orbitofrontal and ventromedial prefrontal cortices. Furthermore, the change in outcome hedonics correlated with activity in the periacqueductal grey (PAG) of the descending pain modulatory system (DPMS). The context manipulation also significantly increased functional connectivity between reward circuitry and the PAG, consistent with a functional change of the DPMS due to the altered motivational state. The findings of this study point to a role for brainstem and reward circuitry in a context-induced “hedonic flip” of pain.

## Summary

When moderate pain was presented in a context of intense pain, it induced a relief and ‘hedonic flip’ such that pain was reported as pleasant.

## 1 Introduction

Pain from nociception is considered intrinsically aversive [42]. Nonetheless, anecdotal evidence suggests that even strong noxious stimulation is sometimes pleasurable, as for spicy food or in the case of sexual masochism [37]. Pleasure and pain are often represented as opposites within a hedonic spectrum [7,13,48], and can have mutually inhibitory effects [36]. These feelings induce competing motivational states that alter the function of the descending pain modulatory system (DPMS) [18]. In line with the view of pain and pleasure as opposites, we have previously shown that safety from pain causes a pleasant feeling of relief [34]. Furthermore, we and others have demonstrated that relief from pain activates reward and valuation circuitry such as the ventral striatum and the ventromedial prefrontal/orbitofrontal cortices (vmPFC/OFC) [3,35,53].

It is well established that neural activity often reflects the *relative* rather than the *absolute* value of events across different contexts [52]. For instance, losing money usually causes negative feelings. However, in a context where all alternative outcomes are larger losses, losing a small amount can elicit positive emotions (relative relief) and activation in ventral striatum and vmPFC/OFC [11,29,41,43,61]. Similarly, macaque orbitofrontal neurons encoded the preferred reward in a reward context, and encoded relative safety (no stimulus) in an aversive context in which the alternative outcome was electric shock [23].

The present study investigated the effects of relative relief on hedonic and physiological reactions to moderate pain. We used a context manipulation to alter the relative value of a moderately painful stimulus. In the control context, the alternative outcome was nonpainful warmth. Thus, the moderately painful stimulus was the worst possible outcome, akin to how pain is commonly perceived in laboratory and real-life settings. In contrast, in the “relative relief context,” the alternative outcome was an intensely painful stimulus. The moderately noxious stimulus, which was identical across the two contexts, was therefore the better of the two possible outcomes and represented relative relief. This design ensured that the moderate pain stimuli in the two contexts were matched for surprise as well as intensity, timing, and frequency of nociceptive input. We measured the hedonics, skin conductance response, and functional magnetic resonance imaging (fMRI) signal associated with moderate pain across these two contexts in 16 healthy volunteers.

We hypothesised that the context manipulation would result in a *hedonic flip*, such that the normally aversive, moderate pain would elicit more positive hedonics and higher activity in reward circuitry including the ventral striatum and vmPFC/OFC. Consequently, since reward system activity inhibits pain [22,36,72], we expected diminished skin conductance and fMRI signal responses within key regions of the brain’s pain network [1,2]. We hypothesised that this relief-related analgesic response would recruit the DPMS (eg, [9,59,64,70,71]). Finally, we expected the relative relief context to induce an affectively and motivationally distinct state in participants. Since the function of the DPMS is state dependent, we hypothesised that this state change would be reflected in alterations in the connectivity pattern of the DPMS, specifically the periacqueductal grey (PAG) [18,19].

## 2 Methods

### 2.1 Participants

Seventeen right-handed healthy volunteers (mean age 25 years, age range 19-41 years, 8 females) were recruited for this study. All participants gave written informed consent, and none had contraindications to MRI. The study was approved by the Central Oxfordshire Clinical Research Ethics Committee (C02.286) and conforms to the guidelines of the Declaration of Helsinki (1996). Participants were reimbursed 25 GBP. One participant’s dataset was incomplete due to technical difficulties. This participant was excluded from analysis, yielding a final n = 16.

### 2.2 Study design

**Fig. 1:**
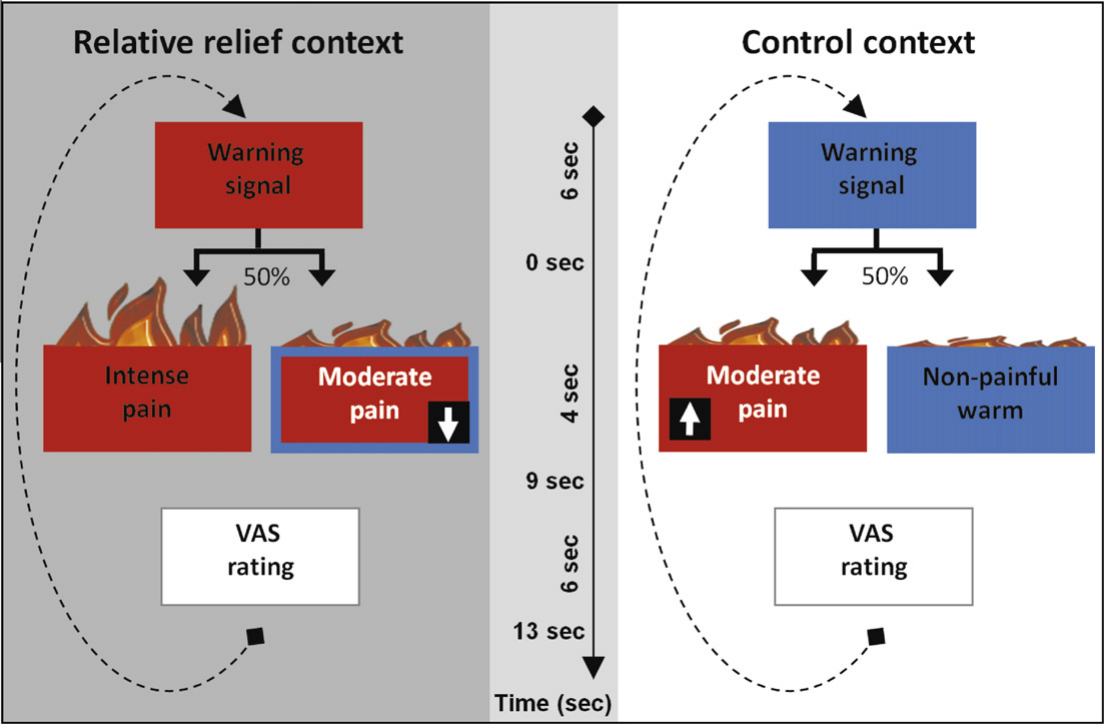
Experimental design. The same moderately painful stimulus was presented in two different contexts (sessions). In the relative relief context, participants were cued to expect a high proportion of intensely painful stimuli. They were informed that in some instances, the warning signal would be replaced by an arrow pointing down, indicating that a lower temperature would be applied to the skin (relative relief). In the control context, participants were cued to expect nonpainful warm stimuli, but were informed that the warning signal would sometimes be followed by a higher temperature, as indicated by an arrow pointing upwards. Thus, the moderate pain stimulus was the worst possible outcome in the control context, akin to how pain is commonly perceived in laboratory and real-life settings. The moderately noxious stimulus was identical across the two contexts, its temperature selected to elicit moderate pain in each individual before the scan. In both contexts, the moderate pain stimuli were 50% probable, and session and stimulus presentation orders were counterbalanced. VAS, visual analogue scale.

The study design is outlined in Fig. 1. The study consisted of two functional MRI scanning sessions of 15 minutes each, separated by a 10-minute structural MRI scan. The order of the two sessions (relative relief session and control session) was counterbalanced across participants. Each session consisted of 24 trials, each lasting ∼36 seconds. Each trial consisted of: 1) a 50% predictive visual cue presented for 6 seconds; followed immediately by 2) heat stimulation for 4 seconds; and finally, 9 seconds after heat offset, 3) a 6-second rating period, where a visual analogue scale (VAS) was presented. After a 13-second interval, another trial began with a new presentation of the visual cue. This interval ensured that the onset of each trial was jittered with respect to the 3-second repetition time (TR). Trial order was pseudorandomised within each session. Before the scan, participants viewed a slideshow explaining what would occur during the study.

In the control session, we used the predictive visual cue (warm cue) to induce expectation of innocuous heat. The warm cue consisted of a green screen and white text (“Warm stimulus coming up…”). In 50% of the trials, the warm cue remained on the screen after the 6-second anticipation period, and during the 4-second innocuous warm thermal stimulation. In the remaining trials, the warm cue was replaced after 6 seconds by a black screen with a white arrow pointing upwards (up arrow), signaling the occurrence of a higher temperature, noxious outcome (the “control moderate”). This visual cue co-occurred with a thermal stimulus calibrated to induce moderate pain.

Similarly, in the relative relief session we presented a predictive cue (intense cue) to induce expectation of intense pain. The intense cue consisted of a red screen and white text (“Heat stimulus coming up…”). In 50% of trials, the intense cue was displayed for 10 seconds, the last 4 of which coincided with a thermal stimulus calibrated to cause intense pain. In the remaining trials, the intense cue was replaced after 6 seconds by a black screen with a white arrow pointing down (down arrow), indicating the lower temperature stimulus (the “relative relief moderate”). As in the control session, this visual cue co-occurred with the moderate pain stimulus (the same temperature as used in the control session).

This study was designed to address alterations in pain perception elicited by the occurrence of a moderately painful stimulus, when this stimulus was associated with relative safety (relief). Previous research showed that moderately painful stimuli are experienced as more painful when the participants expected to receive intense pain (and less painful when they expected mild pain; [2,27,31]). In contrast, the present design always included *correct* visual information about the nature of the stimulus. This information was presented at the onset of each moderate heat stimulus, which replaced the expectation cue.

We used two in-house thermal resistors [8,12,66] to deliver noxious thermal stimuli (4 seconds at destination temperature) to the volar aspect of the participants’ left arm. For each participant, we determined 3 temperatures corresponding to verbal pain intensity ratings of “non-painful warm,” “moderate pain,” and “intense pain” before the start of the experiment proper, but inside the scanner. The mean temperatures used in the experiment were 39.4 ± 1.9, 48.9 ± 2.6, and 53.3 ± 2.8°C (mean ± SD) for the warm, moderate, and intense pain stimulation, respectively.

### 2.3 Hedonic ratings

Hedonic ratings were given as a discrete rating after each heat event. Participants moved a mechanical slider along a visual analogue scale (VAS) to indicate their response. The “outcome hedonics” scale assessed the affect associated with the stimulus occurrence, considering the alternative outcome (“How did you feel about this outcome?”; anchors: negative – positive). The “sensation hedonics” scale assessed the pain or pleasure elicited by the innocuous warm, moderately noxious heat and intensely noxious heat (“What did this sensation feel like?”; anchors: very painful – very pleasant).

The two scales were designed to measure different aspects of the stimulus-induced experience. When presented with the outcome hedonics scale, participants were asked to report on the affective reaction elicited by their *knowledge* of the current outcome, given their knowledge of alternative outcomes. In contrast, for the sensation hedonics scale, participants were instructed to assess how painful or pleasant the *sensory* experience of the stimulus was. The anchors of the sensation hedonics scale were based on a hedonic continuum using Bentham’s definitions, where all aversive feelings are classified as painful and all positive hedonic feelings fall into the category of pleasure [7]. The use of the two scales was explained in detail in the slideshow that participants viewed before the scan. The neutral point was clearly marked at the midpoint of both scales. Therefore, participants were able to differentiate between the negative and positive portions of the scales. The VAS scales were displayed without visible numbers. Ratings on the VAS line were translated into numbers on 11-point scales ranging from −5 to +5, with the neutral point set to 0. The order of presentation of the two rating scales was pseudorandomised within each session.

After each scanning session, participants were asked to report, *on average*, how much dread they associated with the cue in each context (“How much dread did you feel when you saw the stimulus cue?”; anchors: no dread – intense dread). They also reported average pain intensity of each stimulus type on a VAS scale (“How painful was the [intense/warm/unexpected] stimulus?”; anchors: not painful – intensely painful).

### 2.4 Skin conductance

Skin conductance was recorded at 100 Hz using a data acquisition device (PowerLab 8, AD Instruments, Colorado Springs, CO, USA) and dedicated software (Chart 5, AD Instruments). The MRI-compatible galvanic skin response∗ electrodes were filled with skin conductance gel (Henley’s Medical, Welwyn Garden City, Hertfordshire, UK) and secured to the third and fifth digits of the same hand.

### 2.5 MRI data acquisition

Functional imaging data were acquired in a 3 Tesla human MRI system (1 m bore; Oxford Magnet Technology, Oxford, UK) using a birdcage radio frequency coil for pulse transmission and signal detection by a reduced bore gradient coil (Magnex SGRAD MK III, Oxford, UK). A gradient echo-planar imaging sequence with TR = 3 seconds, matrix = 64 × 64; echo time = 30 ms, 41 × 3 mm axial oblique slices, volumes = 323 (the first 4 were “dummy” scans), field of view = 192 × 192, and voxel size = 3 × 3 × 3 mm was used. Functional scans were acquired continuously throughout each of the two 15-minute sessions, which were separated by a ∼10-minute high-resolution structural scan (voxel size 1 × 1 × 1 mm) acquired to improve registration to standard space.

### 2.6 Analysis of hedonic ratings

Statistical analysis of behavioural data was performed using SPSS 12.0 (SPSS Inc, Chicago, IL, USA). VAS scores for the sensation and outcome hedonics scales and postscan ratings of pain intensity and dread were averaged across participants and assayed for significance using paired *t*-tests. Pearson’s correlation test was used to test for significant correlations between the (normally distributed) behavioural measures. The difference in outcome hedonics between the control and relative relief contexts was defined as the “relative relief effect.” This measure was used in subsequent analyses relating behavioural data to functional imaging data.

### 2.7 Analysis of skin conductance data

Chart 5 (AD Instruments) was used for initial processing (low-pass filter, 0.1 Hz) of the skin conductance data. Skin conductance responses were computed for the 6-second period following the stimulus cue in each session (anticipation period) and for the 12-second period following the onset of the heat stimuli (stimulation period) using Matlab 8 (The Mathworks Inc, Natick, MA, USA). Skin conductance data for each event was normalised by subtracting the value of the first time point in each event from the subsequent time points. The peak value from each normalised event was used in the analysis. Four datasets were incomplete due to technical difficulties and were excluded from this analysis.

### 2.8 Analysis of fMRI data

fMRI data analysis was performed in a multistage process using FEAT (functional MRI [fMRI] Expert Analysis Tool) Version 5.92, part of FSL (Oxford Centre for Functional Magnetic Resonance Imaging of the Brain [FMRIB, Oxford, UK] Software Library; http://www.fmrib.ox.ac.uk/fsl). Prestatistics processing was applied as follows: motion correction using MCFLIRT [25]; nonbrain removal using Brain Extraction Tool [54]; spatial smoothing using a Gaussian kernel of full-width-half-maximum 5 mm; and highpass temporal filtering (Gaussian-weighted least-squares straight line fitting, with sigma = 50.0 s). Individual independent component analyses using Probabilistic Independent Component Analysis [6] as implemented in MELODIC (Multivariate Exploratory Linear Decomposition into Independent Components) were applied to each of the preprocessed fMRI datasets (two per participant). The time courses for the independent components that corresponded to spikes and other artefacts (“ring-shaped” activation around edges of the brain, saw-tooth timecourse pattern, and spatial activation patterns corresponding to white matter) were regressed out of the fMRI data using the fsl_regfilt utility (see [26]). The resulting denoised 4-dimensional datasets were used in the general linear model (GLM) analyses.

A unique input stimulus function was defined for each visual cue type (intense and warm), for each heat stimulation type, and for the VAS rating intervals. Input stimulus functions were convolved with the gamma haemodynamic response function (HRF) (mean lag 6 seconds and full-width-at-half-height 6 seconds) to yield regressors (4 per session) for the GLM. The estimated motion parameters for each participant were included as covariates of no interest to reduce spurious activations due to head motion and scanner drift, thereby increasing statistical sensitivity. Time-series statistical analysis was carried out using FMRIB’s Improved Linear Model (FILM) with local autocorrelation correction [68]. Registration to high-resolution structural and standard Montreal Neurological Institute space images was performed using FLIRT [24,25].

Higher-level (group) statistical analysis was performed using FLAME 1 & 2 (FMRIB’s Local Analysis of Mixed Effects) [5,67], which produced *Z* (Gaussianised T/F) statistic images. Only voxels of *Z* > 2.3 were further submitted to cluster-based correction (*P* < 0.05) [69] for multiple voxel-wise comparisons (correcting for multiple comparisons). The following analyses were performed at the group level.

First, we used the contrast (stimulus > rest) to assess the main effect of each stimulus type: intense pain, moderate pain in the relative relief and control contexts, nonpainful warm stimulation, and the warning signal in each of the two contexts. On the basis of these contrasts, we ran a manipulation check that assessed stimulus-related blood-oxygen-level-dependent (BOLD) responses to intense, moderate, and nonpainful heat in a subset of regions within the brain’s pain network [1]. We created 8-mm spheres around the peak activation coordinates in the dorsal anterior cingulate cortex (dACC), bilateral insula, and cerebellum (MNI coordinates 6, 10, 34; ±46, 4, 6; and −6, −46, −26, respectively, taken from an independent, recent investigation of pain [2]). We based this analysis on coordinates from Atlas et al. because of extensive similarities in experimental design, notably the use of a similar range of noxious stimuli and of nonpainful cues alerting participants to the identity of the noxious stimulus [2]. To illustrate that the BOLD signal within these a priori, independently defined regions of interest increased as the stimulus intensity (heat/temperature) increased from nonpainful warm to moderate pain and intense pain, we extracted mean% change values for each region and analysed these using a repeated-measures analysis of variance (linear contrast).

To assess whether these regions of the brain’s pain network would show reduced activity to moderate pain in the relative relief context, we ran a paired *t*-test (control moderate > relative relief moderate) restricted to the above regions of interest (small volume correction). The purpose of this illustrative analysis was to assess the effects of the context manipulation in a selection of key pain-related regions. We based the analysis on peak coordinates selected from an independent, recent study employing a similar range of painful stimuli and cues to investigate the brain mechanisms that link experience with expectation for pain [2]. For a more complete understanding of the role of the brain’s pain network in the context effect, we also conducted a whole-brain analysis using this contrast. Furthermore, a whole-brain paired *t*-test on the opposite contrast (relative relief moderate > control moderate) was performed to identify any regions showing significantly higher activation during moderate pain in the relative relief context.

Next, we identified voxels that showed a significant, positive correlation with the effect of the context manipulation on the hedonic outcome ratings (“relative relief effect”). This analysis enabled the identification of regions that were affected more by the context manipulation in those participants who reported a greater change in pain hedonics in the relative relief context. On the basis of extensive literature implicating the PAG of the brainstem pain modulatory circuitry in the up- and downregulation of pain, this analysis was also run using small volume correction to restrict the analysis to voxels within a 6-mm sphere centred around the PAG (MNI coordinates 6, −30, 14; see, eg, [9,16,17,64]).

As a final analysis step, we investigated whether the context manipulation altered the functional connectivity of the descending pain modulatory system. We used the voxels from the PAG identified in the above analysis as seed voxels. Individual timeseries from these voxels (the entire session timeseries) for each session and participant were added to each existing first-level GLM. Since the PAG and other pain network brain regions were significantly more activated during intense pain in the relative relief session than during nonpainful warm in the control session, the timeseries regressor was orthogonalised with respect to these stimulus types. A statistical map was calculated for each session for each participant. A paired *t*-test was conducted at the group level to identify regions showing a greater covariation with the brainstem seed region throughout the relative relief context compared to the control session (and vice versa) [21].

## 3 Results

### 3.1 Manipulation check

**Fig. 2:**
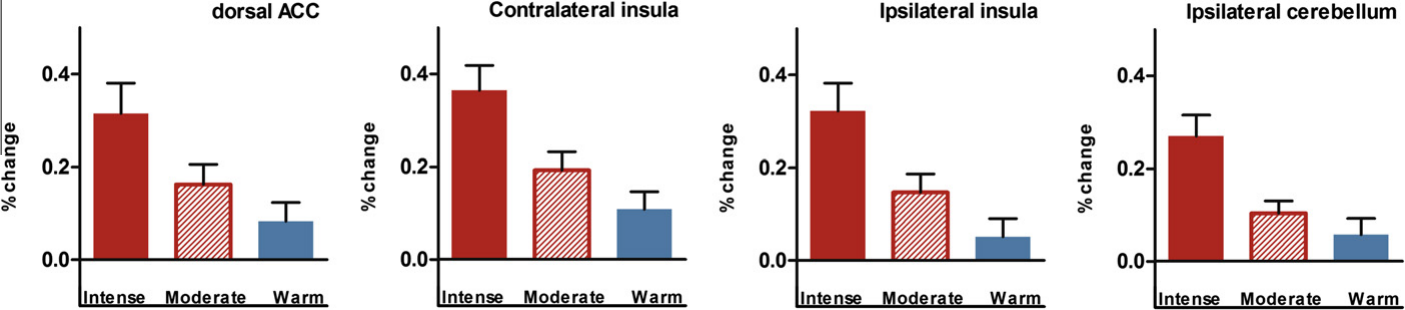
A region of interest analysis using a priori defined areas of the brain’s pain network shows the increase in blood-oxygen-level-dependent (BOLD) signal in the dorsal anterior cingulate (dACC), insula, and cerebellum as the stimulus intensity (heat) increased from nonpainful warm to moderate pain and intense pain. Error bars represent SEM.

First, we assessed the hedonic ratings of the stimuli calibrated to induce intense pain and no pain, respectively. As expected, the intense pain stimulus elicited negative hedonics on both rating scales (outcome hedonics, −2.6 ± 1.9; sensation hedonics, −4.0 ± .9, mean ± SD). The opposite was found for the nonpainful warm stimulus, which elicited positive outcome hedonics (2.6 ± 1.4) and positive sensation hedonics (2.6 ± 1.6). An initial analysis of activity within key regions of the brain’s pain network confirmed that % signal change within the dACC, bilateral insula, and cerebellum (defined a priori from an independent study [2]) was highest during intense pain, lower during moderate pain, and lowest during innocuous warm stimulation (repeated-measures analysis of variance, linear contrast, all *P*s < 0.005, see Fig. 2).

### 3.2 The effect of relative relief on pain hedonics

**Fig. 3:**
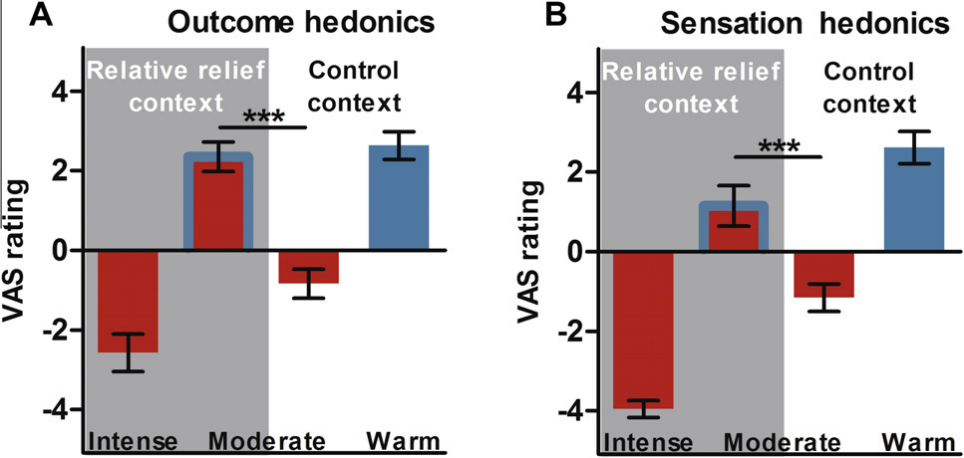
Effects of relative relief on moderate pain hedonics. (A) Moderate pain in the control context elicited negative ratings on the outcome hedonics scale. In contrast, the identical stimulus presented in the relative relief context yielded positive affect. In fact, this positive reaction was of a similar magnitude as the positive response to the nonpainful warm stimulus in the control context. (B) Similarly, when participants focused on rating the sensation hedonics, the control moderate stimulus yielded ratings in the painful range. Paradoxically, given the intrinsic aversiveness of pain, the mean rating of relative relief moderate stimulation was within the *pleasant* range of the sensation hedonics scale (0-5). Error bars represent SEM. VAS, visual analogue scale.

The relative relief moderate stimulus elicited significantly more positive hedonic ratings on both the outcome and sensation hedonics scales (both *P*s < 0.001). In fact, ratings on both scales indicated a *hedonic flip*, such that hedonic feelings towards moderately painful stimulation shifted from negative in the control context to positive in the relative relief context (Fig. 3). Specifically, moderate pain in the control context (“control moderate”) elicited negative hedonic feelings on the outcome hedonics scale (−0.8 ± 1.5, mean ± SD). This stimulus was also reported as painful on the sensation hedonics scale (−1.2 ± 1.4). In contrast, moderate pain in the relative relief context was associated with *positive* hedonic feelings (2.3 ± 1.5) and the average rating fell within the *pleasant* range of the sensation hedonics scale (1.1 ± 2.0). The difference in outcome hedonics between the two contexts (“relative relief effect”) significantly correlated with the difference in sensation hedonics (*r* = 0.70, *P* = 0.003). In other words, the participants who reported more positive affect due to the *knowledge* of the moderate outcome in the relative relief context also rated the sensation elicited by the moderate noxious stimulus as the most pleasant. As expected, dread associated with the warning signal was higher in the relative relief context (5.7 ± 3.0) than in the control context (2.7 ± 2.5; *P* = 0.005). A *t*-test of postsession average pain intensity ratings revealed that the context manipulation caused a significant reduction in perceived intensity of the moderately painful stimulus in the relative relief context (control moderate 5.3 ± 2.4; relative relief moderate: 3.4 ± 2.9; *P* = 0.023). Pain intensity ratings for the intense pain stimulus were, on average, 8.9 ± 1.0, whereas the nonpainful warm stimulus elicited a mean rating of 1.0 ± 2.0.

### 3.3 The effect of relative relief on pain processing and skin conductance

**Supplementary Figure 1:**
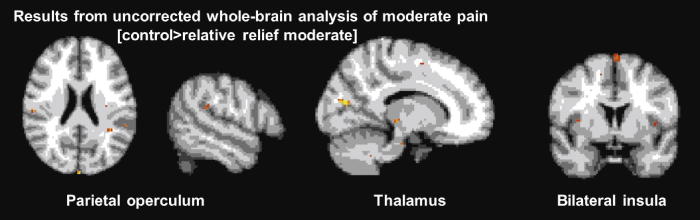
This document contains supplementary figure.

**Fig. 4:**
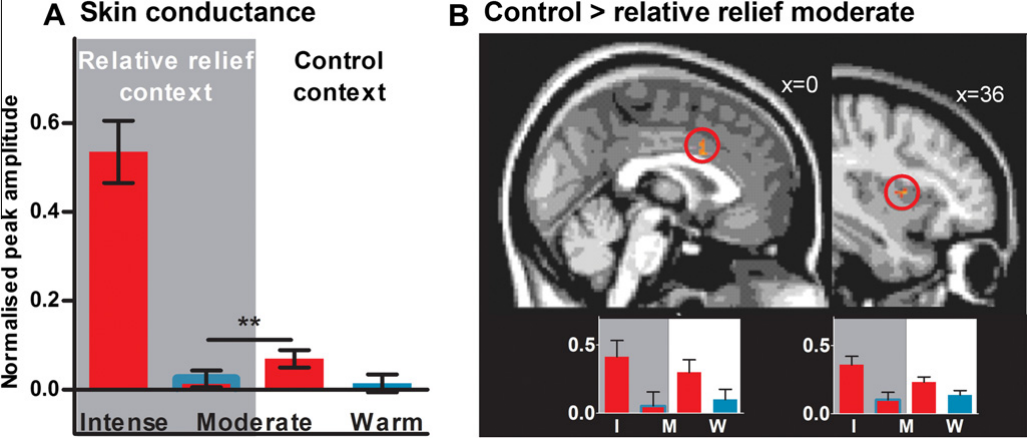
Physiological measures revealed significant differences between moderate stimuli in the relative relief vs control contexts. (A) The measured change in skin conductance during moderate noxious stimulation was significantly lower during moderate pain in the relative relief context than in the control context (*P* < 0.01). (B) Activity in key regions of the brain’s pain network (dorsal anterior cingulate cortex [dACC] and contralateral mid-insula) was significantly reduced during moderate pain in the relative relief context. Functional magnetic resonance imaging statistical maps were thresholded using *Z* > 2.3, *P* < 0.05 corrected, and are shown overlaid on the standard MNI brain. For illustration purposes, bar charts of the mean signal change from the thresholded maps are displayed below the magnetic resonance images, and the right-most image is thresholded at *Z* > 2.3, uncorrected. Error bars represent SEM.

We assessed the effect of the context manipulation on brain activity within the regions of the brain’s pain network used in our initial analyses above (dACC; bilateral insula; cerebellum). Voxels in the dACC and contralateral insula showed significantly reduced activity during moderately painful stimuli in the relative relief context compared to moderate pain in the control context (Fig. 4). No voxels were significantly active after correction for multiple comparisons in a whole-brain analysis of this contrast. At a more lenient threshold of *Z* > 2.3 uncorrected, voxels in the bilateral parietal operculum, thalamus, and ipsilateral insula also showed a reduction due to the context manipulation (Supplementary Fig. 1). We found a similar context-dependent reduction in skin conductance to the relative relief moderate stimulus compared to the control moderate (paired *t*-test, *P* = 0.009; Fig. 4). Skin conductance measures are thought to be sensitive to the threat value of a stimulus. Together, the reduction in BOLD signal in key areas of the brain’s pain network and the significant reduction in pain-related autonomic response found here corroborate the subjective reports of a reduction in negative pain hedonics and the postsession reports of reduced pain intensity.

### 3.4 Activation of reward and brainstem pain modulatory circuitry during relative relief

The paired *t*-test (relative relief moderate > control moderate) revealed significantly higher activation in vmPFC areas, including rostral and subgenual anterior cingulate and medial OFC during moderate pain in the relative relief condition. vmPFC regions have been consistently shown to encode the positive hedonic value of rewards, including the relative reward of monetary loss or safety from punishment [23,44,52,57,58,60].

**Fig. 5:**
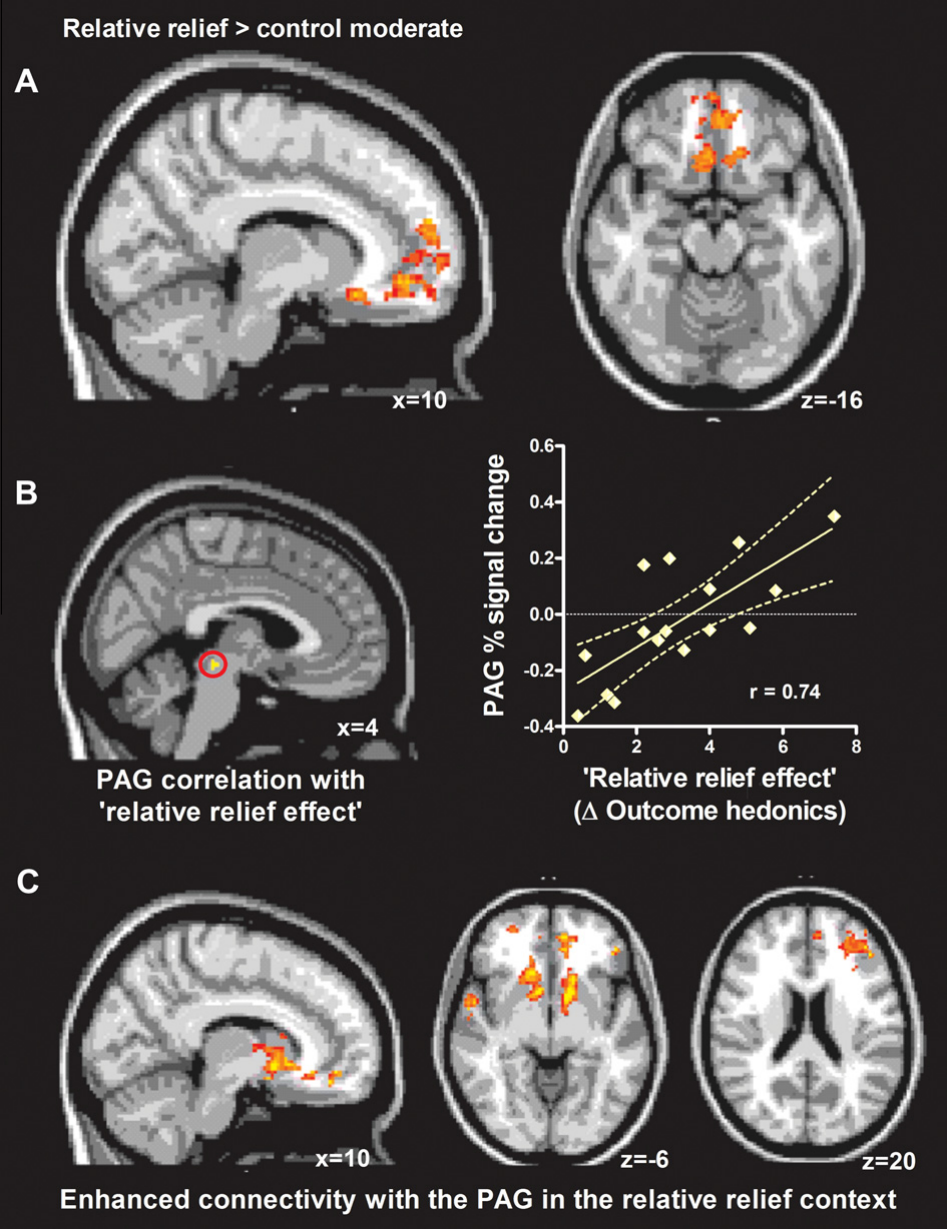
Role of reward and brainstem pain modulatory system in relative relief. (A) Ventromedial prefrontal, medial orbitofrontal cortex, and rostral cingulate cortices showed significantly higher activation during relative relief moderate stimulation compared to the control moderate. These regions have typically been associated with reward and analgesia processing. (B) The relative relief effect correlated with the between-session difference in blood-oxygen-level-dependent (BOLD) signal in the periacqueductal grey (PAG) during moderate pain stimulation. (C) A functional connectivity analysis comparing the PAG’s covariation pattern throughout the relative relief session with the covariation pattern throughout the control session revealed significantly higher connectivity between the PAG and reward/valuation circuitry, including ventral striatum, ventromedial prefrontal cortex, and ventrolateral prefrontal cortex in the relative relief session.

We also assessed the role of the brainstem pain modulatory circuitry (DPMS), specifically the PAG, to determine the role of this powerful endogenous pain modulatory system for the relative relief effect. A paired *t*-test comparing activity in the PAG during the relative relief and the control moderate stimuli revealed no significant differences. However, voxels in the PAG correlated significantly and positively with the reported hedonic “relative relief effect.” The participants who showed the largest positive change in outcome hedonics also activated this region of the DPMS more during moderate pain in the relative relief context than in the control context (Fig. 5).

### 3.5 Altered brainstem pain modulatory circuitry connectivity in the relative relief context

In a final analysis step, we investigated how the context manipulation affected the functional connectivity of the PAG during the relative relief session. As explained by Fields [19], the function of the brainstem pain modulatory circuitry changes according to motivational state, that is, depending on the presence of potential rewards or dangers. For instance, the presence of a reward can lead to a motivational state in which pain sensitivity is reduced due to activation of OFF cells in the rostroventromedial medulla. Here, the relative relief context indicated a higher risk of intense pain and greater relief during relative safety. We found that vmPFC, left ventrolateral PFC, and bilateral ventral striatum showed significantly greater correlation with the PAG during the relative relief session, as compared to the control session. This improved functional connectivity between reward circuitry and the PAG throughout the high-threat, high-relief context is consistent with a change in motivational state during this context. No brain regions showed significantly higher connectivity with the PAG in the opposite contrast (control session > relative relief session).

## 4 Discussion

The results from this study demonstrate that when experienced in a context of intense pain, a moderately noxious stimulus can elicit positive hedonic feelings. Since moderate pain in this context represented relative relief, we hypothesised that healthy volunteers would report significantly *more positive affect* in response to this outcome. Not only did our results confirm this prediction, but more surprisingly, participants rated the moderately noxious stimulus as *pleasant* in the relative relief context. Despite anecdotal evidence that pain can elicit pleasure in certain specific contexts, to our knowledge this is the first controlled laboratory demonstration of such a *hedonic flip*.

Importantly, participants’ hedonic ratings were corroborated by changes in physiological responses to pain. As expected, pain-related skin conductance response, which is sensitive to the salience or threat value of a stimulus (eg, [10,39]), was attenuated when moderate pain represented relative relief. Similarly, BOLD responses in dorsal anterior cingulate and contralateral insula, key regions of the brain’s pain network [1,2], were significantly reduced compared to the moderately painful stimulus in the control context. Moreover, relative to the control stimulus, moderate pain in the relative relief context showed higher activation in vmPFC regions, consistent with the increased hedonic value of this stimulus [29,40,43,46,51,61].

A series of previous studies have found evidence that the ventromedial prefrontal and orbitofrontal cortices encode hedonic value. This part of the reward system responds to the stimulus that represents the best possible outcome in a given context, be it monetary gain [30,40,51], a small loss in the context of large losses [29,43,61], or safety from aversive (but nonpainful) shocks [23,35,53]. The present results demonstrate that when moderately painful stimulation represents the best possible outcome, it similarly increases activation in these value-encoding regions. Importantly, this “relative relief effect” was elicited by presenting moderate pain in two separate sessions. Electrophysiological data from macaque OFC neurons suggest that the context effect is only present *within* each context or session [44]. Therefore, a neutral outcome is encoded as a reward when the only alternative outcome within the context is aversive [23], but not when both rewards and punishments are presented within the *same* context [44]. Based on these findings, we predict that the context effect documented in the present study would be reduced or even abolished, if the intense pain, the moderate pain, and the nonpainful warmth were presented intermixed in one session.

In general, and with certain exceptions such as the relative relief context created in the present study, moderate pain represents an aversive outcome. Pain and pleasure typically fall on opposite sides of the hedonic continuum [7], and inhibit each other [15,20,28,32,36,49,50,55,56,62]. Pain and pleasure also induce competing motivational states. High-reward contexts can induce an appetitive state in which pain is downregulated, since it is adaptive to ignore pain and obtain the reward. Aversive contexts can cause both up- and downregulation of pain [19]. The present findings demonstrate that it is nevertheless possible to pair pain with a rewarding outcome (relief from intense pain) in a laboratory setting.

In the present study, participants gave their ratings on bipolar scales representing a hedonic continuum between negative and positive, pain and pleasure. Note that these rating scales are not designed to measure mixed emotions (eg, see [33]). Thus, it is possible that the context manipulation resulted in mixed feelings of pleasure *and* pain. The advantage of the bipolar hedonic scales, on the other hand, is that participants report the *sum total* of their hedonic feelings (eg, see [48]). This method is therefore more relevant for understanding the relationship between hedonic feelings and behaviour, mirroring the *one* behavioural decision (usually to approach reward or avoid pain) that must be selected when motivations for pain and pleasure compete [45].

The moderate stimulus in the control context represented the worst possible outcome and followed a visual cue signalling nonpainful stimulation. Since phasic moderate pain can often occur unexpectedly (eg, stubbing a toe) and typically represents the worst possible outcome, this aspect of the design has ecological validity. If the perception of moderate pain in the control context were modulated by a perception of “relative danger” or disappointment related to the worst outcome, we would expect strongly negative hedonic ratings to this stimulus. Instead, ratings on both hedonic scales revealed only weak negative hedonics to the control moderate.

Both skin conductance and fMRI results corroborate the *hedonic flip* reported on the bipolar rating scales. In the relative relief context, we found a significant reduction in pain-related BOLD signal in dACC and contralateral insula. Reduced activity in this subset of the brain’s pain network has been reported in several previous studies of endogenous analgesia [4,63,65]. Interestingly, the results indicate that the skin conductance response to moderate pain reflected the *relative* and not the absolute value of this stimulus. Skin conductance measures are sensitive to changes in salience rather than hedonic value *per se*. Our results indicate that the salience of moderate pain was significantly higher when it represented the worst possible outcome. In contrast, the salience of moderate pain in the relative relief context was reduced to the same level as that of nonpainful stimulation in the control context.

Based on previous literature on reward-related and other types of endogenous analgesia [9,14,22,36,47,70], we expected to find recruitment of the PAG in response to the context manipulation. While we found no significant main effect of context in the PAG, our investigation of the role of this brainstem nucleus for the “relative relief effect” revealed a significant, positive correlation between the PAG and the improvement in hedonic feelings in the relative relief context. Participants who showed the strongest context-induced improvement in subjective feelings towards moderate pain also showed larger PAG responses in this context, compared to the control moderate. The PAG is a complex structure involved in a range of pain- and non-pain-related processes. Our result supports the interpretation that the DPMS is involved in the context-induced “hedonic flip” in subjective ratings.

An important feature of the DPMS is that its function depends on the context, specifically, the motivational state of the organism [18,19]. The PAG was previously shown to exhibit enhanced functional connectivity during pain with primary sensorimotor cortices, thalamus, cerebellum, and other brainstem sites [38]. This pattern of functional connectivity differs markedly from the enhanced vmPFC–PAG connectivity commonly involved in analgesia [9,17,64,70], which has been shown to be opioid dependent [17]. In the present study, we found evidence for increased connectivity of the vmPFC, ventral striatum, and left ventrolateral PFC with voxels in the PAG in the relative relief context. Unlike the above-mentioned studies, which reported enhanced connectivity during pain only, we found that connectivity between these regions and the PAG was enhanced for the entire session. This finding likely reflects a change in affective and motivational state due to the context manipulation. Compared to the control session, the relative relief context was associated with increased relief responses, high likelihood of intense pain, and an increase in reported dread of pain.

The context manipulation employed here altered both the range of possible outcomes and the specific expectation elicited by the warning signal prior to thermal stimulation. The two moderately painful stimuli were equally probable, and the onset of each moderate stimulus co-occurred in time with a visual cue informing participants that the temperature applied was either lower (relative relief context) or higher (control context) than the preceding visual warning signal had indicated. This design ensures equal surprise and salience of moderate pain in each context, but it does not allow us to disentangle the specific contribution of expectation from that of the range of possible outcomes. Similarly, the effect of seeing the visual cue that indicated a lower or higher temperature cannot be disentangled temporally from the effect of feeling the moderate stimulus. Previous studies using heat pain have shown that ratings of moderate pain increase when intense pain is expected [2,27,31], possibly due to participants’ difficulty in quickly assessing the identity of the thermal stimulus. The visual cue used here was necessary to alert participants to the correct identity of the stimulus. However, to disentangle the realisation that the current outcome is the best option, from the pain perception itself, the relative relief visual cue must be separated from the painful stimulus temporally, as in [35].

In conclusion, we have shown that by altering the context in which moderate pain is experienced, it is possible to *invert* pain hedonics from negative to positive. This context manipulation altered autonomic responses and activity in insula and dACC, implying a mechanism of reward-induced analgesia. The increased activity in vmPFC regions and the positive association between brainstem PAG and the subjective hedonic effect, suggest a putative mechanism through which the relative relief context modulates the evaluation of moderately noxious stimulus. The present paradigm that illustrates our capacity to hedonically flip the experience of pain, provides a useful tool for further investigation of alternative relief processes in clinical populations where pain cannot be avoided.

## Conflict of interest statement

The authors have no conflicts of interest to report.
